# From Microstructure to Shade Shift: Confocal and Spectrophotometric Evaluation of Peroxide-Induced Dental Bleaching

**DOI:** 10.3390/jcm14134642

**Published:** 2025-07-01

**Authors:** Berivan Laura Rebeca Buzatu, Magda Mihaela Luca, Atena Galuscan, Adrian Ovidiu Vaduva, Aurora Doris Fratila, Ramona Dumitrescu, Ruxandra Sava-Rosianu, Octavia Balean, Roxana Buzatu, Daniela Jumanca

**Affiliations:** 1 Translational and Experimental Clinical Research Centre in Oral Health, Department of Preventive, Community Dentistry and Oral Health, University of Medicine and Pharmacy “Victor Babes”, 300040 Timisoara, Romania; berivan.buzatu@umft.ro (B.L.R.B.); galuscan.atena@umft.ro (A.G.); sava-rosianu.ruxandra@umft.ro (R.S.-R.); balean.octavia@umft.ro (O.B.);jumanca.daniela@umft.ro (D.J.); 2 Clinic of Preventive, Community Dentistry and Oral Health, “Victor Babes” University of Medicine and Pharmacy, Eftimie Murgu Sq. no 2, 300041 Timisoara, Romania; 3 Department of Pediatric Dentistry, Faculty of Dental Medicine, “Victor Babes” University of Medicine and Pharmacy Timisoara, Eftimie Murgu Square 2, 300041 Timisoara, Romania; luca.magda@umft.ro; 4 Department of Pathology, Victor Babeş University of Medicine and Pharmacy, 300041 Timişoara, Romania; 5 Faculty of Dental Medicine, Ludwig Maximilian University of Munich, Goethestrasse 70, 80336 Munich, Germany; a.fratila@campus.lmu.de; 6 Department of Dental Aesthetics, Faculty of Dental Medicine, “Victor Babes” University of Medicine and Pharmacy Timisoara, Revolutiei Boulevard 9, 300041 Timisoara, Romania; roxana.buzatu@umft.ro

**Keywords:** tooth bleaching, confocal laser scanning microscopy (CLSM), spectrophotometric analysis, hydrogen peroxide, carbamide peroxide

## Abstract

**Background/Objectives**: Tooth bleaching is a widely requested aesthetic procedure in modern dentistry. However, the structural effects of high-concentration peroxide-based bleaching agents on enamel remain insufficiently understood. This study aims to evaluate and compare the effects of three in-office bleaching agents—Opalescence Boost (40% hydrogen peroxide [HP]), Opalescence Quick (45% carbamide peroxide [CP]), and BlancOne Ultra + (35% hydrogen peroxide [HP])—on enamel surface characteristics and color change using confocal laser scanning microscopy (CLSM) and spectrophotometric analysis. **Methods**: Forty-two extracted human teeth were sectioned and divided into experimental and control halves. Each experimental specimen underwent bleaching according to the manufacturer’s protocol. Color measurements were conducted at baseline, immediately post-treatment, at 3 days, 7 days, and 6 months following treatment using the Vita Easyshade^®^ spectrophotometer. Color differences were calculated using the CIEDE2000 (ΔE_00_) formula. Enamel surface morphology was assessed by CLSM. Data were analyzed using Wilcoxon and Kruskal–Wallis tests (*p* < 0.05), performed with SPSS v23. **Results**: All bleaching agents produced clinically perceptible color changes (ΔE_00_ > 3.3). Opalescence Boost achieved the highest and most consistent whitening effect (mean ΔE_00_ > 11), while Opalescence Quick showed moderate efficacy (ΔE_00_ ~6–8), and BlancOne Ultra+ induced milder changes (ΔE_00_ ~4–5). CLSM imaging revealed surface alterations in all bleached samples, with more pronounced changes observed in specimens treated with higher peroxide concentrations. **Conclusions**: All three bleaching systems were effective in improving enamel color, with Opalescence Boost delivering the most substantial and durable effect. CLSM analysis confirmed morphological changes in enamel without evidence of severe damage. These results underscore the importance of selecting bleaching protocols that balance efficacy with enamel safety. Further in vivo studies are recommended to validate long-term structural effects and support clinical decision-making.

## 1. Introduction

In today’s social media-driven culture, the appearance of a bright and white smile has become increasingly valued, often symbolizing health, confidence, and attractiveness. The aesthetic appearance of teeth, closely linked to perceptions of health and beauty, has made cosmetic dentistry a fundamental aspect of modern restorative practice [1,2]. A bright, well-maintained smile is often associated with positive social impressions, psychological well-being, and overall satisfaction in personal interactions [3]. Dental bleaching is a widely performed procedure in clinical practice, designed to improve tooth color, thereby enhancing aesthetic outcomes and patient satisfaction [4]. The effectiveness of bleaching treatments is influenced by several factors, including the type of bleaching agent used, the pH of the oral environment, the application protocol, and the nature of the tooth discoloration [5].

The growing demand for tooth bleaching has led to the development of a wide range of whitening options. These products vary from professionally administered in-office whitening (OW) and dentist-supervised at-home whitening (HW) to over-the-counter (OTC) treatments and do-it-yourself (DIY) methods, which are used without professional supervision [6]. Among the various whitening methods available, peroxide- and carbamide-based bleaching agents remain the most commonly used. However, their impact on dental ultrastructure is still not fully understood, requiring further investigation to assess their potential effects on enamel integrity and long-term safety [7].

Tooth whitening has evolved significantly in recent years, including various techniques beyond peroxide-based gels. Phosphoric acid etching, primarily used for enamel conditioning, also enhances peroxide penetration by increasing enamel permeability, although overuse can lead to demineralization and increased sensitivity [8]. Halogen curing lamps have been employed to activate bleaching agents via thermal stimulation, yet concerns remain regarding pulp overheating and soft tissue discomfort due to their non-uniform light dispersion and heat generation [9]. In contrast, laser-assisted bleaching, especially with diode lasers (e.g., 445–970 nm), has demonstrated accelerated bleaching reactions and reduced chair time, provided energy output is controlled to avoid pulpal temperature increases above 5.6 °C—a threshold associated with irreversible pulpal damage [10,11]. Simultaneously, the field of dental materials has seen a shift toward bioactive and antimicrobial compounds. As highlighted by Yudaev & Chistyakov (2024) [12], recent developments focus on integrating natural ingredients (e.g., flavonoids, plant extracts, propolis) in dental composites, adhesives, and mouthwashes to reduce cytotoxicity and resist microbial colonization. These materials offer a promising alternative to synthetic agents, enhancing safety and therapeutic outcomes across restorative, orthodontic, and endodontic applications. Together, these innovations in whitening technology and material science not only address aesthetic demands but also aim to optimize biocompatibility, antimicrobial efficacy, and patient comfort in modern dentistry.

Hydrogen peroxide (HP) is the most widely used bleaching agent in tooth whitening treatments, often applied directly or in the form of carbamide peroxide (CH_6_N_2_O_3_). Due to their low molecular weight, peroxides readily diffuse into enamel and dentin, penetrating through enamel prisms. As hydrogen peroxide infiltrates the enamel, it dissociates into peroxide anions (HO_2_^−^) and oxygen free radicals, collectively referred to as reactive oxygen species (ROS). These ROS initiate an oxidation process that breaks down pigmented organic molecules into smaller, colorless inorganic compounds, ultimately leading to a whitening effect [13]. Carbamide peroxide requires chemical activation, decomposing into approximately 30% hydrogen peroxide and 70% urea. The choice of bleaching agent concentration varies depending on the application method [14]. Low-concentration hydrogen peroxide gels (4% to 22%) are typically used in at-home whitening treatments, requiring multiple applications over time, whereas high-concentration gels (25% to 40%) are primarily reserved for in-office procedures, offering more immediate and controlled results under professional supervision [15].

Vital tooth bleaching has become increasingly popular in recent years as a simple, cost-effective, and minimally invasive method for treating tooth discoloration. Since its introduction, it has been widely recognized as an effective and straightforward aesthetic procedure for eliminating both intrinsic and extrinsic stains from teeth [16]. Intrinsic staining may be influenced by genetic predisposition, antibiotic use during tooth development (such as tetracycline-induced staining), or excessive fluoride exposure leading to fluorosis [17]. In contrast, extrinsic discoloration occurs due to the adsorption of pigments on the tooth surface, often caused by frequent tobacco use, consumption of pigmented foods and beverages, and inadequate oral hygiene leading to biofilm accumulation [17].

These pigments, known as chromophores, absorb light within the visible spectrum and reflect wavelengths associated with yellowish or brownish hues [18]. Organic compounds responsible for discoloration originate from common dietary sources such as tea, coffee, and red wine. Their molecular structures contain carbonyl or aromatic groups, which form stable bonds with dental biofilm and calculus [17,18]. The removal of chromophores is achieved through the oxidation of organic compounds by bleaching agents, primarily HP, which serves as the active ingredient in most whitening systems [19]. Hydrogen peroxide is highly soluble in water and exhibits an acidic pH in solution. Due to its unpaired electrons, it possesses strong reactivity and an oxidative capacity capable of breaking down both organic and inorganic molecules [6]. Bleaching occurs through the breakdown of peroxide into free radicals, which then interact with large pigment molecules, breaking them down into smaller, less pigmented compounds. This oxidation process, primarily driven by HP, affects a wide range of organic substances [16].

The tooth whitening process involves prolonged or high-concentration exposure of bleaching agents directly to the tooth surface. This has raised concerns about their potential adverse effects on tooth structure [20]. Literature reports potential effects on dental hard tissues following bleaching, including mineral loss, increased susceptibility to erosion and caries, heightened surface roughness, reduced enamel tensile strength, diminished fracture resistance, and decreased abrasion resistance [16].

Accurate color assessment is essential in biomimetic dentistry, as it directly influences clinical outcomes, research quality, and overall patient satisfaction [21,22,23]. To enable objective and reproducible color evaluation, the International Commission on Illumination (CIE) introduced the CIELAB color space [24], which has become a standard in dental research and practice [23,25]. This system defines color using three parameters: L* for lightness [23], a* for the red-green axis, and b* for the yellow-blue axis [25,26]. Color differences between two points within this space can be quantified using the ΔE value, which serves to determine whether variations are perceptible or clinically acceptable.

Confocal laser scanning microscopy (CLSM) has emerged as a powerful tool for non-invasive analysis of dental hard tissues, providing high-resolution imaging and three-dimensional reconstructions without requiring physical sectioning. Unlike conventional optical and fluorescence microscopy, which can be affected by out-of-focus light, CLSM enhances image clarity by eliminating interference from non-focal planes. This technique utilizes a focused laser beam and selective detection system, enabling precise visualization of structural and morphological changes in enamel and dentin. In dentistry, CLSM has been widely applied to assess enamel defects, mineralization patterns, and the penetration of bleaching agents, offering a detailed evaluation of microarchitectural alterations. Given its ability to provide real-time, high-contrast imaging at a microscopic level, CLSM represents an essential diagnostic and research tool [27].

Given the increasing demand for cosmetic dentistry, assessing the structural effects of carbamide and hydrogen peroxide-based bleaching agents is essential for optimizing both treatment efficacy and safety. While widely used, these whitening agents may alter enamel microstructure, requiring detailed investigation. Utilizing CLSM imaging and 3D reconstructions, this study examines surface microtopography, defect depth, and enamel prism organization, comparing bleached and unbleached samples. Additionally, it explores the correlation between bleaching concentration, exposure time, and structural changes, providing valuable insights for refining whitening protocols and ensuring the long-term preservation of enamel health.

This study aims to assess the effects of different in-office bleaching protocols on enamel surface characteristics, following the ISO 28399:2021 guidelines [28], which define safety and performance standards for external tooth whitening products. The research focuses on three commonly used bleaching agents—Opalescence Quick (45% CP), Opalescence Boost (40%HP), and BlancOne Ultra+ (35% HP)—each applied according to specific clinical protocols. To evaluate their impact on enamel integrity, the study employs confocal laser scanning microscopy (CLSM) to investigate changes in mineral content, surface morphology, and demineralization potential, ensuring a standardized approach to understanding the effects of professional whitening treatments.

## 2. Materials and Methods

This study included extracted human molars and premolars obtained for clinical reasons, in compliance with ethical guidelines and approved by the Bioethics Committee of the Victor Babeș University of Medicine and Pharmacy, Timișoara (Approval No. 09/11.03.2024). Conducted in accordance with the Declaration of Helsinki and Good Practice in Biomedical Research, the study ensured rigorous specimen selection.

### 2.1. Sample Size

The sample size for this study was determined to ensure statistical validity and reliable comparisons between the control and experimental groups. Each extracted tooth was sectioned into two equal halves, with one half serving as the control and the other as the bleached specimen.

The minimum required sample size was calculated using G*Power 3.1 software, based on an expected effect size of 0.5, a significance level (α) of 0.05, and a statistical power of 0.80, in line with prior studies assessing enamel structural changes following bleaching treatments. This analysis indicated that a total of 42 specimens (n = 14 per group) would be sufficient to detect significant differences. To account for potential variability or specimen loss during preparation, an additional 20% was initially considered, increasing the target sample pool to approximately 50 teeth. However, no specimens were damaged or excluded throughout the study process. As a result, all 42 prepared specimens were included in the final analysis. This approach is consistent with previous in vitro investigations that apply an over-recruitment margin to ensure statistical robustness while maintaining feasibility under controlled laboratory conditions [29,30].

Extracted human molars and premolars obtained from an anonymized biobank were used for this study, following strict ethical guidelines. After extraction, the teeth were thoroughly cleaned of soft tissue debris by gently scraping the external surface with periodontal instruments. To preserve their structural integrity and maintain hydration, the specimens were stored in artificial saliva for no longer than one month before the experimental procedures. This approach ensures that the enamel properties remain as close as possible to their in vivo state, reducing potential biases related to dehydration or alterations in mineral content. The calculated sample size aligns with previous in vitro studies [29] assessing enamel alterations post-bleaching, ensuring robust statistical analysis while maintaining feasibility in laboratory conditions. The teeth were examined under ×10 magnification (OMS2356, Zumax Medical Co, Ltd., Suzhou, Jiangsu, China) and those with cracks, caries, dental restorations, or structural enamel defects were excluded.

### 2.2. Specimen Preparation

A total of 42 teeth were selected for the study. After removing calculus deposits, the teeth were stored in 0.1% thymol solution for five days. Sectioning was performed 2 mm above the cementoenamel junction using a diamond saw under water cooling. Each crown was sectioned along the cervical-occlusal axis to obtain two equal halves. Both halves were assigned the same identification number, with one serving as an experimental specimen and the other as a control (each experimental half had a corresponding control half from the same tooth).

Each specimen was embedded in a self-cure acrylic resin (UNIFAST Trad, GC America) and polished using Soft-lex discs (Soft-lexTM, 3M ESPE, St. Paul, MN, USA) with grit sizes of 42 μm, 30 μm, and 15 μm for two minutes per stage, followed by rinsing with distilled water. To simulate intraoral conditions and maintain enamel hydration throughout the experimental timeline, all specimens were stored in artificial saliva at 37 °C in a biological incubator. This storage protocol was initiated immediately after extraction and maintained continuously throughout specimen preparation, bleaching procedures, and all analysis intervals—including prior to spectrophotometric and CLSM evaluations—to prevent dehydration and preserve both optical and structural enamel properties. The artificial saliva solution was renewed every 48 h to ensure chemical stability and avoid contamination. The formulation included sodium bicarbonate (2190 mg), potassium phosphate (1270 mg), magnesium chloride (125 mg), calcium chloride (441 mg), potassium chloride (820 mg), sodium fluoride (4.5 mg), nipazole (100 mg), nipagin (10 mg), sorbitol (24 mg), carboxymethylcellulose (8 mg), and distilled water (1000 mL), adjusted to a pH of 7.0. The solution was manipulated in a local compounding pharmacy, following the standardized protocol described by Vilhena et al. [31].

### 2.3. Macroscopic Visualization of Dental Samples Used in the Study

**Figure 1:**
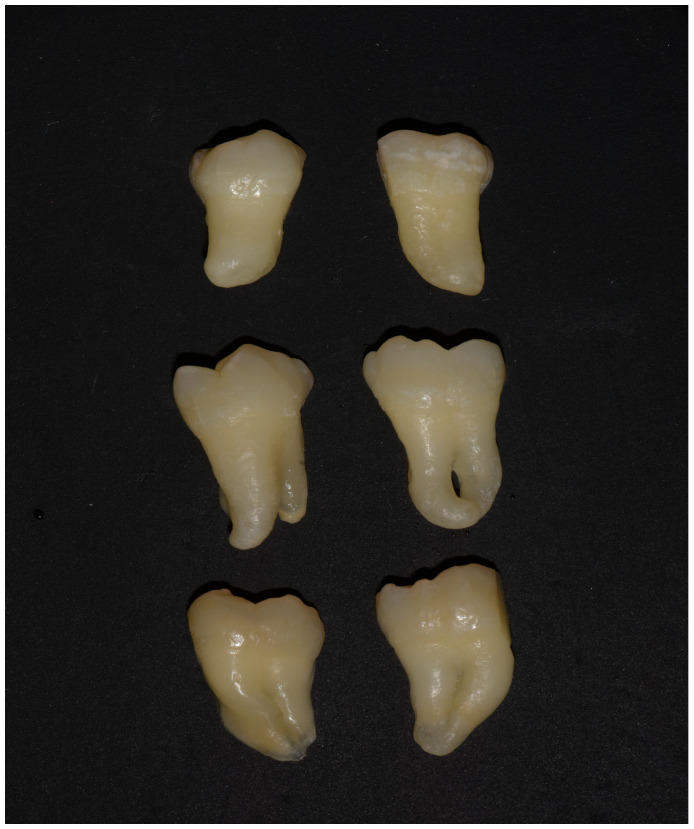
Macroscopic view of extracted human teeth prior to sectioning.

Figure 1 displays six freshly extracted human premolars and molars prior to sectioning. Calculus deposits were removed, and the specimens were photographed before being sectioned 2 mm above the cementoenamel junction, as described in the methodology. These intact crowns were later divided along the cervical–occlusal axis to obtain two symmetrical halves—one designated as a control and the other for experimental bleaching treatment. This step ensured matched comparisons within the same anatomical structure.

### 2.4. Bleaching Procedure

The experimental group consisted of extracted teeth treated with three different in-office bleaching agents commonly used in clinical practice: Opalescence Quick (45% carbamide peroxide [CP], Ultradent Products Inc., South Jordan, UT, USA), Opalescence Boost (40% HP, Ultradent Products Inc., South Jordan, UT, USA), and BlancOne Ultra+ (35% HP, IDS, Italy). Each bleaching protocol followed the manufacturer’s recommendations.

- Opalescence Quick (Ultradent Products Inc., South Jordan, UT, USA) contains 45% carbamide peroxide (a slower-acting bleaching compound that decomposes into hydrogen peroxide and urea. The gel is white, ready to use, and does not require mixing or light activation, making it suitable for short, in-office procedures. Opalescence Quick (45% PC, Ultradent, USA) was applied directly to the enamel surface in a 1–2 mm layer for 15–30 min per session, without light activation.
- Opalescence Boost (Ultradent Products Inc., South Jordan, UT, USA) is a chemically activated in-office bleaching agent containing 40% hydrogen peroxide (HP). It is supplied in a dual-syringe system that ensures fresh mixing of the peroxide gel immediately prior to application. The product is applied without the need for light activation and has a characteristic red color. Opalescence Boost (40% HP, Ultradent, South Jordan, UT, USA) was applied in a 1–2 mm layer, with one application lasting 20 min, two applications totaling 40 min, and three applications up to 60 min per session (not exceeding three applications per session), without light activation.
- BlancOne Ultra+ (IDS Spa, Savona, Italy) is a professional in-office whitening system based on 35% HP, supplied in powder form within a mixing container. The bleaching agent requires manual preparation by adding and mixing the provided H_2_O_2_ solution with the powder. The final product has an orange color and is intended for immediate application after activation. BlancOne Ultra+ (35% HP, IDS, Italy) was applied in a 1–2 mm layer, with light activation for 8–10 min per application, totaling three applications per session.

Prior to bleaching, the enamel surfaces were mechanically cleaned to remove organic debris and rinsed with distilled water. Representative enamel specimens were selected and positioned on microscope slides without sectioning, in order to allow for surface-level analysis under the confocal laser scanning microscope. Details regarding the bleaching agents used are provided in Table 1.

### 2.5. Confocal Microscopy Evaluation

The specimens were examined using an Olympus Fluoview FV1000 confocal laser scanning microscope equipped with a UPLSAPO 10× objective lens (numerical aperture NA 0.40). Laser excitation was performed at 635 nm, and emitted light was detected at 647 nm. High-resolution Z-stack images were acquired at 1600 × 1600 pixels, with a slice interval of 1.2 μm, allowing for 3D reconstruction using Bitplane Imaris v7.4 software. The specimens were examined using an Olympus Fluoview FV1000 confocal laser scanning microscope equipped with a UPLSAPO 20× objective lens (numerical aperture NA 0.75). Laser excitation was performed at 635 nm, and emitted light was detected at 647 nm. High-resolution Z-stack images were acquired at 1600 × 1600 pixels, with a slice interval of 1.2 μm, a scanning speed of 2 μs/pixel, and 65% laser transmissivity. The photomultiplier tube (PMT) voltage was set to 629 V to optimize signal detection and contrast. This imaging setup enabled three-dimensional reconstruction using Bitplane Imaris v7.4 software. CLSM imaging revealed distinct variations in enamel surface morphology across different bleaching protocols [32]. CLSM imaging revealed distinct variations in enamel surface morphology across different bleaching protocols.

### 2.6. Spectrophotometric Shade Evaluation

A spectrophotometer (Vita Easyshade^®^ V Compact, Vita Zahnfabrik, Bad Säckingen, Germany) was employed to objectively assess tooth color changes [33]. The measurements were conducted under standardized conditions, with a spectral range of 360–750 nm, a wavelength interval of 10 nm, and a 45° reflectance geometry. All assessments were performed against a black background, and the final result for each sample represented the average of three consecutive scans.

Color measurements were performed using the CIE Lab* color space, as defined by the International Commission on Illumination (CIE) [34]. In this system, L* represents lightness, a* indicates the red-green axis, and b* represents the yellow-blue axis. Additionally, C* refers to chroma, and h° denotes hue. In our analysis, ΔL and Δb were calculated as the difference between bleached and control specimens (Δ = bleached − control). Therefore, negative ΔL values indicate increased lightness (whitening), and negative Δb values reflect a reduction in yellow chroma, both of which are consistent with an effective whitening process. Measurement was taken approximately at the center of each sample, with the device probe (0.5 mm diameter) positioned perpendicularly to the surface. To ensure measurement accuracy and reproducibility, the spectrophotometer was calibrated at the beginning of each session using the reference calibration block supplied by the manufacturer [35], in accordance with the standards traceable to the National Institute of Standards and Technology (NIST). Color differences between timepoints were calculated using the CIEDE2000 (ΔE_00_) formula, as proposed by Sharma et al. (2005) [36], which incorporates perceptual corrections for lightness, chroma, and hue.

The CIEDE2000 (ΔE_00_) formula was selected due to its improved agreement with human visual perception compared to earlier models such as CIE76 or CIE94. The ΔE_00_ equation incorporates weighting functions and a rotation term to better reflect perceptual uniformity across the color space. The formula is defined as follows:

$$\Delta E2000=\sqrt{{\left(\frac{\Delta L^{\prime }}{K_{L}\cdot S_{L}}\right)}^{2}+{\left(\frac{\Delta C^{\prime }}{K_{C}\cdot S_{C}}\right)}^{2}+{\left(\frac{\Delta H^{\prime }}{K_{H}\cdot S_{H}}\right)}^{2}+R_{T}\cdot \left(\frac{\Delta C^{\prime }}{K_{C}\cdot S_{C}}\right)\cdot \left(\frac{\Delta H^{\prime }}{K_{H}\cdot S_{H}}\right)}$$

where ΔL′, ΔC′, and ΔH′ are the differences in lightness, chroma, and hue, respectively; S_L_, S_C_, and S_H_ are the corresponding weighting functions; k_L_, k_C_, and k_H_ are the respective parametric correction terms; and R_T_ is a rotation function accounting for chroma–hue interaction. This formulation has become the standard in dental research for color stability and perceptibility analysis [36].

To quantify the perceived differences between control and bleached specimens, color changes were calculated using the CIEDE2000 (ΔE_00_) formula, which has been shown to better reflect visual perception than the traditional CIELAB (ΔE*ab) metric in dental applications [37]. Perceptibility and acceptability thresholds were interpreted in accordance with published standards in esthetic dentistry. According to Ghinea et al. (2010) [38], the 50:50% perceptibility threshold for ΔE_00_ is approximately 1.30, while the 50:50% acceptability threshold is 2.25. For broader interpretative purposes, color changes were further categorized using a five-level scale as follows:

- ΔE_00_ < 1: not perceptible;
- 1 ≤ ΔE_00_ < 2: perceptible by experts only;
- 2 ≤ ΔE_00_ < 3.3: perceptible by trained observers;
- 3.3 ≤ ΔE_00_ < 5: clinically visible difference;
- ΔE_00_ ≥ 5: strong and easily perceptible difference.

Color measurements were performed at five timepoints: baseline (prior to treatment), immediately after bleaching, at 3 days, 7 days, and 6 months post-treatment. Before each measurement, specimens were rinsed with distilled water and gently dried to avoid interference from surface residues. Throughout the study period, all specimens were stored in artificial saliva at 37 °C, with the solution renewed every 48 h, in order to preserve enamel hydration and closely mimic intraoral conditions between shade assessment intervals.

### 2.7. Statistical Analysis

Statistical analysis was conducted using SPSS^®^ software, version 23 (SPSS Inc., Chicago, IL, USA). Nonparametric tests were applied to compare experimental samples with their corresponding control samples. The Wilcoxon signed-rank test was applied to compare color differences (ΔE_00_) between control and bleached specimens within each group. The Kruskal–Wallis test was used to assess differences in ΔE_00_ among the bleaching agents at each timepoint. When statistically significant differences were detected, Dunn’s post-hoc test with Bonferroni correction was performed to determine which groups differed significantly. A significance level of *p* < 0.05 was considered statistically significant. A significance level of *p* < 0.05 was considered statistically significant.

## 3. Results

### 3.1. Confocal Microscopy Evaluation

**Figure 2:**
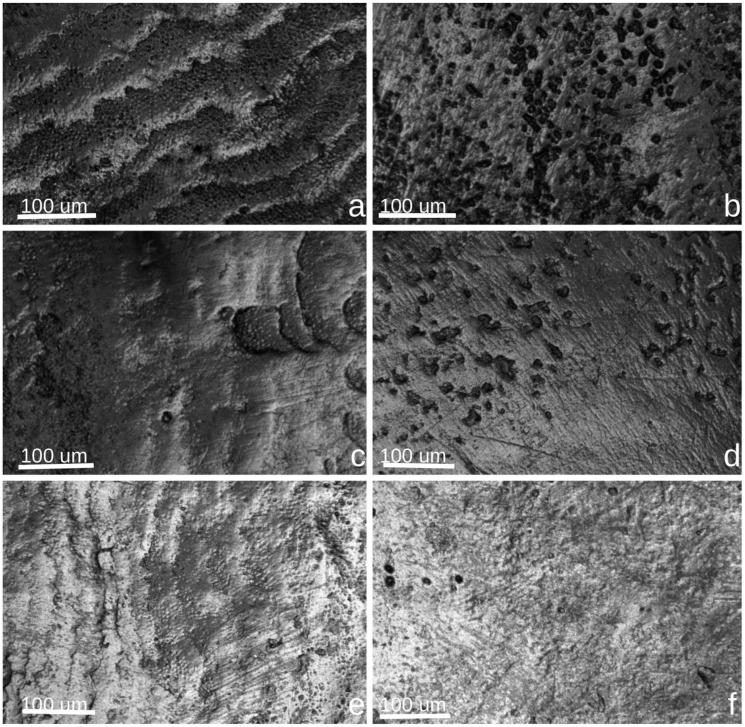
Confocal laser scanning microscopy images of enamel surfaces before and after bleaching treatments. (**a**) Control enamel treated with Opalescence Quick (CP 45%); (**b**) enamel after bleaching with Opalescence Quick (CP 45%); (**c**) control enamel treated with Opalescence Boost 40% HP; (**d**) enamel after bleaching with Opalescence Boost 40% HP; (**e**) control enamel treated with BlancOne Ultra+ 35% HP; (**f**) enamel after bleaching with BlancOne Ultra+ 35% HP.

Confocal microscopy analysis revealed distinct surface alterations in enamel following exposure to the different bleaching agents. Control specimens (Figure 2a,c,e) showed smooth, intact enamel morphology with well-defined structural features and minimal surface disruption. After bleaching, the enamel treated with Opalescence Quick (b) exhibited evident surface roughness and micro-porosities, indicating superficial mineral loss. The enamel surface in the Opalescence Boost group (d) showed more pronounced irregularities, with crater-like depressions and a heterogeneous texture, consistent with the stronger oxidative activity of 40% hydrogen peroxide. In contrast, enamel treated with BlancOne Ultra+ (f) demonstrated the least morphological change, maintaining a relatively uniform and compact surface, with only minor texture alterations. These findings suggest a variable impact on enamel topography depending on the concentration and formulation of the bleaching agent used. These observations are based on qualitative evaluation of CLSM images and were not supported by quantitative image analysis.

### 3.2. Evaluation of Color Changes Through Spectrophotometric Shade Analysis

Table 2 presents the mean color differences (ΔE_00_ CIEDE2000) between control and bleached enamel for each bleaching agent at four timepoints: immediately after treatment, 3 days, 7 days, and 6 months post-bleaching. Among the tested agents, Opalescence Boost (40% HP) consistently showed the most pronounced whitening effect, with mean ΔE values exceeding 11 at all stages and peaking at 13.94 at 3 days. This sustained high performance suggests a rapid and stable bleaching effect over time. Opalescence Quick (45% CP) demonstrated moderate efficacy, with ΔE values ranging from 5.85 immediately post-treatment to 8.56 at 7 days, and remaining relatively stable at 6 months (8.55). These values indicate a visibly perceptible but less intense whitening outcome compared to hydrogen peroxide-based agents. BlancOne Ultra+ (35% HP) resulted in the lowest ΔE values, consistently around 4–5 across all timepoints. Although these changes surpass the clinical perceptibility threshold (ΔE > 3.3), they reflect a milder and more conservative whitening effect.

A Kruskal–Wallis test revealed no statistically significant differences between groups at baseline or at 3 and 7 days (*p* > 0.05). However, a statistically significant difference was observed at 6 months (χ^2^ = 6.25, *p* = 0.044), indicating divergence in long-term color stability.

**Figure 3:**
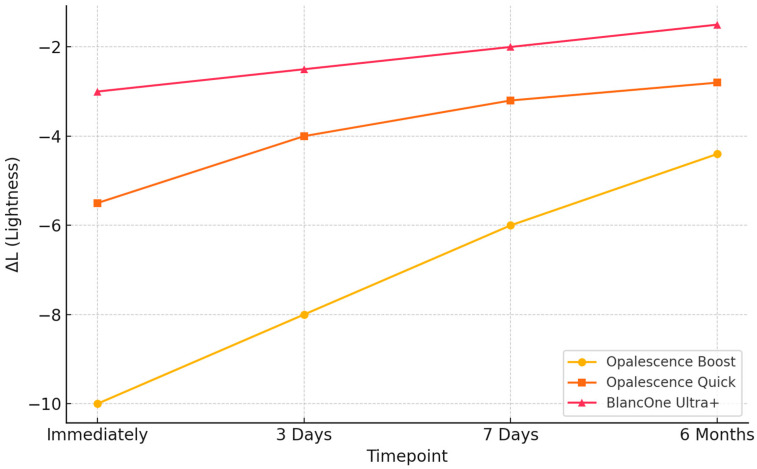
Longitudinal change in enamel lightness (ΔL) after bleaching. ΔL was calculated as bleached—control; thus, more negative values indicate increased lightness (whitening).

**Figure 4:**
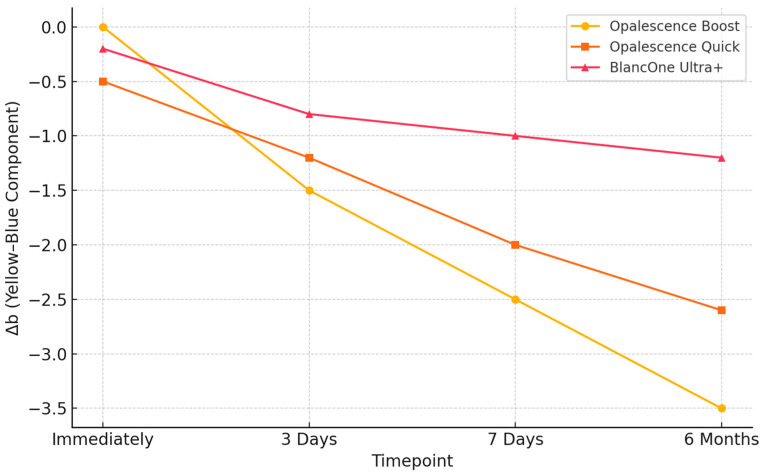
Evolution of the yellow–blue component (Δb) in bleached enamel over time. Δb was calculated as bleached—control; negative values indicate a reduction in yellow chroma, suggesting a whitening effect.

In addition to overall color changes, component analysis of ΔL (lightness) and Δb (yellow-blue) was performed to assess the directionality of the whitening effect. These parameters were calculated as the difference between bleached and control specimens (Δ = bleached − control). Accordingly, negative ΔL values indicate increased lightness (whitening), while negative Δb values denote a reduction in yellow chroma—both reflecting effective whitening. Among the tested agents, Opalescence Boost exhibited the most pronounced and sustained changes in both components, maintaining more negative ΔL and Δb values over time. Although BlancOne Ultra+ showed a similar directional trend, the amplitude of changes was smaller, suggesting a milder effect. These findings are illustrated in the Figure 3 and Figure 4.

Regarding the lightness component (ΔL), Opalescence Boost demonstrated the most substantial change, with values starting around −10 immediately post-treatment and gradually increasing to −4.3 at six months, indicating a partial loss of brightness over time. Opalescence Quick followed a similar trend, increasing from −5.5 to −2.5 across the same period. For the yellow–blue component (Δb), all groups exhibited progressive decreases, indicating whitening via chroma reduction. Opalescence Boost reached −3.5 at six months, while Opalescence Quick declined from −0.5 to −2.7. BlancOne Ultra+ showed the least pronounced shifts in both parameters, suggesting a more conservative whitening profile. These changes confirm that the observed ΔE_00_ differences result from genuine whitening effects, as indicated by increasingly negative ΔL values (increased lightness) and decreasing Δb values (reduced yellow chroma), particularly in the Opalescence Boost group. These trends validate the efficacy of hydrogen peroxide-based protocols.

## 4. Discussion

In this study, confocal laser scanning microscopy (CLSM) was used to evaluate structural changes in enamel surfaces following exposure to peroxide-based bleaching agents. CLSM combines optical and computational imaging to produce high-resolution, real-time 3D reconstructions of enamel surfaces. By utilizing a laser source for fluorophore excitation, CLSM enables the visualization of peroxide diffusion through enamel, dentin, and biofilms, generating detailed two-dimensional images that reveal structural alterations. Compared to scanning electron microscopy (SEM) and other histological evaluation methods, CLSM offers distinct advantages, particularly in assessing the depth of penetration and distribution of bleaching agents within dental tissues. Additionally, CLSM has been widely used in microbiological research to quantify bacterial presence within dentinal tubules, demonstrating its versatility as a non-destructive and highly precise analytical tool [39].

The effects of hydrogen peroxide-based bleaching products on enamel remain inconclusive. While some studies report adverse effects, such as decreased microhardness, changes in elastic modulus, and mineral loss, others suggest that these alterations have minimal clinical significance, as enamel can be restored through saliva remineralization or the use of remineralizing agents [40].

The effectiveness and biological impact of bleaching agents are influenced by both their concentration and the duration of application. It has been observed that low-concentration CP-based products, commonly used in at-home bleaching, may induce comparable or even greater enamel alterations than short-duration, high-concentration HP products due to prolonged exposure times [29,41]. Nevertheless, other studies have reported minimal to no significant changes in enamel microhardness at low CP concentrations when proper application protocols are followed [42]. This suggests that both agent concentration and exposure duration must be considered when evaluating the safety and efficacy of bleaching systems.

A systematic review of 55 in vitro studies on enamel microhardness post-whitening indicates that the risk of enamel impairment decreases when experimental conditions closely resemble the oral environment. However, no in vivo studies have been conducted to confirm these findings [40].

Ultrastructural changes in enamel and dentin are influenced by several factors, primarily the pH and concentration of the bleaching agent. Products with a lower pH tend to cause more structural alterations compared to those with a neutral or alkaline pH, even at the same concentration. Similarly, higher concentrations of bleaching agents generally lead to greater structural changes than lower concentrations. However, findings in this area remain controversial [40]. The pH variation of bleaching agents is a significant factor influencing changes in hydroxyapatite (HAp). More acidic pH levels, as observed in certain hydrogen peroxide-based gels, can lead to progressive enamel demineralization, particularly when the bleaching agent is used excessively or beyond the manufacturer’s recommendations [43].

In contrast, carbamide peroxide gels, which have a more basic pH, do not induce HAp dissolution but instead facilitate the oxidation of organic enamel components [44]. The breakdown of carbamide peroxide releases by-products such as urea and ammonia, which disrupt hydrogen bonds in proteins, ultimately weakening the structural support of HAp crystals. Thus, mineral content alterations may result from the penetration of the oxidizing agent into the enamel structure and its subsequent decomposition into reactive oxygen species. These reactive species interact with the inorganic components of HAp crystals, leading to their gradual dissolution [45].

The bleaching agents used in this study had pH values ranging from slightly acidic to neutral (5.6–7.2), thereby minimizing the influence of strongly acidic conditions. This allowed for a more accurate evaluation of peroxide-related effects, independent of the etching potential associated with low pH levels, as previously reported in the literature [40].

Although the current in vitro design did not allow for direct assessment of hypersensitivity, it is important to recognize that high-concentration hydrogen peroxide bleaching agents are frequently associated with transient tooth sensitivity in clinical settings. To mitigate this, clinicians routinely implement preventive strategies such as limiting exposure duration, using neutral pH formulations, spacing treatment sessions, and applying desensitizing agents either before or after bleaching. Among the most effective desensitizing substances are 5% potassium nitrate, sodium fluoride varnishes, and casein phosphopeptide–amorphous calcium phosphate (CPP-ACP) complexes. A recent meta-analysis confirmed that both potassium nitrate and CPP-ACP significantly reduce post-bleaching sensitivity compared to untreated controls [46]. Additional clinical trials have demonstrated that topical application of CPP-ACP (e.g., MI Paste^®^) or fluoride significantly reduces both thermal and tactile sensitivity following in-office procedures, without compromising bleaching efficacy [47,48,49]. These findings underscore the clinical importance of incorporating desensitizing protocols as standard practice in bleaching treatments, particularly when high-concentration agents are employed.

To ensure consistency, each treated specimen had its own untreated control, using two halves of the same tooth. This approach, based on previous methodologies [50], accounts for natural variations in enamel structure, age, and individual characteristics, which could otherwise influence the results. Throughout the treatment period, all samples were stored in artificial saliva to replicate conditions similar to those in clinical practice, maintaining enamel hydration and minimizing external influences.

Both Opalescence Quick (45% carbamide peroxide), Opalescence Boost (40% hydrogen peroxide), and BlancOne Ultra+ (35% HP) induced similar morphological changes on the enamel surface and led to a comparable reduction in mineral content in both enamel and dentin, despite differences in hydrogen peroxide concentration among the products. Notably, dentin and the cementodentinal junction remained unaffected at a structural level, even though all three bleaching agents were applied directly to the dentin. Most CLSM-based studies investigating commercial bleaching products at varying concentrations have reported similar results [40,51,52].

In our study, CLSM qualitatively demonstrated that, regardless of the bleaching agent used, dentinal tubule exposure increased when comparing pre- and post-treatment conditions. These microstructural changes were consistently observed across all treatment groups based on qualitative CLSM evaluation, which revealed increased surface irregularities and apparent dentinal tubule exposure following bleaching. While our study focused on surface morphology and qualitative enamel changes observed through CLSM, we recognize the importance of assessing the depth of peroxide penetration and associated demineralization. Naim et al. [53] conducted a quantitative in vitro evaluation of enamel demineralization depth following bleaching, using fluorescence microscopy to measure lesion depth. Their results demonstrated that structural alterations may extend below the surface and vary depending on peroxide concentration and application duration. Although our methodology did not include depth quantification, future investigations should integrate such measurements to better characterize subsurface enamel response to bleaching agents and improve risk assessment related to long-term enamel integrity [53].

From a clinical standpoint, the enamel microstructural changes observed may have important implications for patient care. Increased surface roughness and porosity can trap acids and dental biofilm, heightening the risk of erosion and caries, particularly in acidic oral environments [54,55,56]. These structural alterations also offer pathways for fluid movement, potentially exacerbating post-bleaching sensitivity [57]. Moreover, compromised enamel surfaces can affect adhesive bonding; numerous studies show a significant reduction in bond strength—up to 60%—when restorations are placed immediately after bleaching, with improvements seen after a delay of 7–21 days [54,57]. These insights emphasize the need for clinical caution: practitioners should consider delaying bonding procedures, advise careful post-bleaching care, and possibly apply remineralizing agents to restore surface integrity and reduce sensitivity.

The present findings confirm that all evaluated bleaching protocols produced clinically noticeable changes in enamel color. Among the tested agents, Opalescence Boost 40% HP resulted in the greatest whitening effect, with high ΔE values maintained throughout the six-month follow-up. In our analysis, ΔL and Δb values were calculated as the difference between bleached and control specimens (Δ = bleached − control). Therefore, negative ΔL values indicate increased lightness (whitening), and negative Δb values reflect a reduction in yellow chroma—both consistent with an effective whitening process. All three bleaching protocols demonstrated directionality consistent with whitening; however, Opalescence Boost exhibited the greatest magnitude of change across both parameters. Specifically, ΔL values for Opalescence Boost reached approximately −10 immediately after treatment, indicating a strong initial increase in lightness. Over the six-month follow-up, these values became less negative (approaching −4 to −6), reflecting a gradual decrease in the whitening effect, though overall brightness remained improved compared to baseline. We acknowledge that more negative ΔL values indicate increased lightness, as ΔL was calculated as bleached—control. Thus, the shift from approximately −10 to −4.4 over time reflects a partial loss of brightness.

BlancOne Ultra+ showed a similar trend, but with smaller initial changes and ΔL values generally ranging from −4 to −5. A consistent pattern was observed for the yellow–blue component (Δb), where all treatment groups exhibited progressive reductions over time, indicating a decrease in yellow chroma. Opalescence Boost produced the most pronounced and sustained effect, reaching −3.5 at six months, followed by Opalescence Quick with a moderate decrease to −2.7. BlancOne Ultra+ showed milder changes throughout the follow-up period. These results reinforce the interpretation that Opalescence Boost achieved the most intense and stable directional whitening effect, although all protocols led to perceptible improvements in tooth color. This outcome suggests strong and durable oxidative potential, supporting the agent’s efficacy in achieving long-lasting esthetic results. In contrast, Opalescence Quick 45% CP demonstrated moderate whitening, while BlancOne Ultra+ 35% HP led to milder, yet still perceptible effects. Notably, Opalescence Quick, containing 45% CP, exhibited a more gradual yet stable whitening trajectory compared to high-concentration hydrogen peroxide agents. Carbamide peroxide decomposes slowly into hydrogen peroxide and urea, ensuring sustained release of reactive oxygen species, which promotes deeper diffusion and more consistent oxidation of chromogenic compounds in enamel and dentin [18]. Additionally, carbamide-based gels often maintain a neutral to slightly basic pH and include stabilizers that help preserve enamel integrity and reduce post-treatment discoloration rebound. Clinical and in vitro evidence supports the notion that this controlled oxidative mechanism results in improved long-term color stability—especially in home-use protocols—while mitigating sensitivity.

The relatively lower impact of BlancOne Ultra+ may be attributed to its reduced peroxide concentration and different activation method. While immediate and short-term differences between products were not statistically significant, a divergence became evident at the six-month mark (*p* = 0.044).

These results are consistent with previous studies [58] emphasizing the influence of hydrogen peroxide concentration on bleaching efficacy, demonstrating that higher peroxide levels result in more significant color changes, supporting the superior whitening performance observed with Opalescence Boost 40% HP. Further investigations are warranted to evaluate the influence of pH, application time, and enamel composition on the long-term outcomes of tooth whitening treatments.

This study represents the first direct comparison of the effects of Opalescence Quick, Opalescence Boost, and BlancOne Ultra+ on enamel structure using confocal laser scanning microscopy (CLSM). The experimental protocol was carefully standardized, with all specimens stored in artificial saliva to simulate intraoral conditions and limit external variables. Through CLSM imaging, the study qualitatively assessed enamel surface changes and dentinal tubule exposure, while quantitative analysis of perimeter and area variations provided further insight into the structural impact of each bleaching agent.

A key methodological strength of this study lies in its use of confocal laser scanning microscopy (CLSM), which enables high-resolution, three-dimensional imaging of enamel surfaces with minimal sample preparation. This non-destructive approach preserves the structural integrity of dental tissues in a hydrated state, allowing for detailed surface and subsurface analysis without compromising specimen quality. Image acquisition was standardized to ensure consistency and reliability across all evaluations. Another important aspect is the simulation of intraoral conditions through the use of artificial saliva, maintained at 37 °C and refreshed every 48 h. This storage protocol supported consistent hydration and minimized extrinsic variability, offering a more realistic environment than previous models based on distilled water. In addition, the split-tooth design—where each tooth served as its own control—enhanced internal validity by controlling for natural variations in enamel structure. Collectively, these methodological choices contribute to the robustness and clinical relevance of the findings.

It is also important to consider the implications of bleaching procedures in teeth with pre-existing structural enamel defects. Molar-incisor hypomineralization (MIH), a condition characterized by hypomineralized enamel with increased porosity and reduced hardness, has been associated with higher susceptibility to caries and mechanical damage. A recent systematic review by Mazur et al. [59] highlighted the clinical challenges posed by MIH, including compromised surface integrity and elevated caries risk. These findings underscore the need for caution when applying bleaching agents in structurally compromised teeth, where the potential for further mineral loss or hypersensitivity may be greater than in sound enamel [59].

Despite the methodological strengths of this study—such as the use of CLSM, standardized bleaching protocols, and controlled sample storage in artificial saliva—several limitations inherent to the in vitro design must be acknowledged. While artificial saliva offers a basic simulation of the oral environment, it lacks the complex enzymatic, immunological, and buffering properties of natural saliva, and cannot fully replicate intraoral dynamics such as salivary flow, thermal fluctuations, mechanical forces (e.g., mastication and brushing), and pH variation. These physiological factors play a crucial role in modulating the bleaching process, enamel demineralization, and subsequent remineralization. Therefore, caution is warranted when extrapolating our findings to clinical practice, and further in vivo studies are needed to validate the observed effects under real-world conditions. Additionally, although color stability was assessed up to six months post-treatment, structural evaluations were limited to the immediate post-bleaching period, without monitoring potential long-term enamel recovery or remineralization. Furthermore, while CLSM provided high-resolution, non-invasive visualization of enamel surface alterations, it does not yield quantitative parameters such as surface roughness values (Ra, Rz) in micrometers. Future investigations could benefit from the inclusion of contact or non-contact profilometric techniques compliant with ISO 4287 standards [60] to obtain precise numerical data and validate CLSM-based observations.

Lastly, to provide a more comprehensive understanding of the impact of bleaching agents, future studies should incorporate mechanical testing (e.g., microhardness, fracture resistance) and complementary imaging modalities such as scanning electron microscopy (SEM) or Raman spectroscopy. These additions would enable a multidimensional analysis of both morphological and functional enamel responses to bleaching treatments.

## 5. Conclusions

This in vitro study demonstrated that all three tested in-office bleaching agents—Opalescence Boost (40% HP), Opalescence Quick (45% CP), and BlancOne Ultra+ (35% HP)—produced clinically noticeable tooth color improvements, with varying levels of efficacy and impact on enamel surface integrity. Opalescence Boost achieved the most significant and long-lasting whitening effect, while BlancOne Ultra+ exhibited a milder yet stable response. CLSM analysis revealed surface changes across all treatments, particularly with higher peroxide concentrations, though no severe damage was observed under controlled conditions. These findings highlight the need to consider both whitening efficacy and structural safety in selecting bleaching agents. Further in vivo investigations are necessary to confirm enamel recovery potential and guide the development of safer, more effective bleaching protocols.

## Figures and Tables

**Table 1:** Composition of the bleaching agents used in the study.

Product Name	Composition	Manufacturer	Lot Number
Opalescence Boost	40% HP pH = 7	Ultradent Products Inc., South Jordan, UT, USA	ULT4754
Opalescence Quick	45% CP pH = 5.6–7.2	Ultradent Products Inc., South Jordan, UT, USA	BWNV9
BlancOne Ultra+	35% HP pH = 6–7.2	IDS Spa, Savona, Italy	00422

**Table 2:** Mean ΔE_00_ CIEDE2000 values (± standard deviation) for each bleaching agent across all timepoints.

Bleaching Agent	Mean ΔE_00_ (±SD) Immediately	Mean ΔE_00_ (±SD) 3 Days	Mean ΔE_00_ (±SD) 7 Days	Mean ΔE_00_ (±SD) 6 Months
Opalescence Boost 40% HP	13.85 ± 4.65	13.94 ± 4.49	11.18 ± 3.70	13.08 ± 2.41
Opalescence Quick 35% CP	5.85 ± 4.18	5.85 ± 4.18	8.56 ± 4.43	8.55 ± 2.23
BlancOne Ultra+ 35% HP	4.03 ± 1.36	4.02 ± 1.28	3.18 ± 4.07	4.67 ± 1.74

## Data Availability

The data presented in this study are available on request from the corresponding author.
